# Supplementary material for: Antibacterial and Anti-Biofilm Activity of Omega-3 Polyunsaturated Fatty Acids against Periprosthetic Joint Infections-Isolated Multi-Drug Resistant Strains
Source: Biomedicines. 2021 Mar 26;9(4):334. doi: 10.3390/biomedicines9040334 (PMC8065983; doi:10.3390/biomedicines9040334)
Supplement: Supplementary file 1 [file biomedicines-09-00334-s001.pdf]

Antimicrobials	Strains							
	S.epidermidis *1457*	*S. aureus* *ATCC 25923*	*P. aeruginosa* *ATCC27853*	*S.haemolytic* *(PI)*	*S.aureus* *(PI)*	*S.simulans* *(PI)*	*S.lugdunensis* *(PI)*	*S.warneri* *(PI)*
Amikacin	S		S	S	S	S	S	S
Aminopenicillin	S	S	-	S	R	R	R	R
Amino+Clav acid	S	S	-	S	S	S	S	S
Azithromycin	-	S	I	S	S	S	S	S
Aztreonam	-	-	-	R	R	R	R	R
Cefazolin	S	S	I	S	S	S	S	S
Cefepime	-	S	I	S	S	S	S	S
Cefotaxime	S	S	-	S	S	S	S	S
Cefoxitin	S	S	-	S	S	S	S	S
Ceftazidime	R	S	-	R	R	R	R	R
Ceftriaxone	S	S	-	S	S	S	S	S
Cefuroxime	S	S	-	S	S	S	S	S
Ciprofloxacin	S	S	I	S	S	S	S	S
Clindamycin	S	S	-	R	S	S	S	S
Ertapenem	S	-	-	S	S	S	S	S
Erythromycin	S	S	-	-	-	-	-	-
Fosfomycin	S	-	-	S	S	S	S	R
Fusidic acid	S	-	-	R	S	S	S	S
Gentamicin	S	S	-	S	S	S	S	S
Imipenem	S	S	I	S	S	S	S	S
Levofloxacin	S	S	-	S	S	S	S	S
Linezolid	S	S	-	S	S	S	S	S
Meropenem	S	S	S	S	S	S	S	S
Moxifloxacin	-	S	-	S	S	S	S	S
Penicillin G	S	S	-	S	R	R	R	R
Piper-Tazob	S	S	I	S	S	S	S	S
Rifampicin	S	S	-	S	S	S	S	S
Tetracyclin	S	S	-	S	S	S	S	S
Tigecyclin	-	-	-	S	S	S	S	S
Trimeth+Sulf	S	S	R	S	S	S	S	S
Vancomycin	S	S	-	S	S	S	S	S

Table 1. – Antibiotic resistance rates of the tested strains. **S**=susceptible; **R**=resistant; **I**=intermediate; (-) non-tested. (Concentration of antibiotic substances in the antimicrobial susceptibility test discs: Amikacin 30 μg; Aminopenicillin 10 μg; Aminopenicillin+Clavulanate acid 20/10 μg; Azithromycin 15 μg; Aztreonam 30 μg; Cefazolin 30 μg; Cefepime30 μg; Cefotaxime 30 μg; Cefoxitin 30 μg; Ceftazidime 30 μg; Ceftriaxone 30 μg; Cefuroxime 30 μg; Ciprofloxacin 5 μg; Clindamycin 2 μg; Ertapenem 10 μg; Fosfomycin 200 μg; Fusidic acid 10 μg; Gentamicin 10 μg; Imipenem 10 μg; Levofloxacin 5 μg; Linezolid 30 μg; Meropenem 10 μg; Moxifloxacin 5 μg; Penicillin G 1 μg; Piperacillin-Tazobactam 100/10 μg; Rifampicin 5 μg; Tetracyclin 30 μg; Tigecyclin 15 μg; Trimethoprim+Sulfamethoxazole 5 μg; Vancomycin 5 μg).
